# *Porphyromonas gingivalis* aggravates alcohol-related liver injury via gut microbiome-HO-1-ACSL4-dependent ferroptosis

**DOI:** 10.3389/fmicb.2025.1554703

**Published:** 2025-04-02

**Authors:** Xuezhe Feng, Yue Wang, Cheng Zhu, Qian Huai, Juanjuan Cui

**Affiliations:** ^1^Department of Stomatology, First Affiliated Hospital of Anhui Medical University, Hefei, China; ^2^Department of Oncology, First Affiliated Hospital of Anhui Medical University, Hefei, China

**Keywords:** Porphyromonas gingivalis, alcoholic liver disease, gut microbiome, ferroptosis, HO-1, ACSL4

## Abstract

**Background:**

Alcoholic liver disease (ALD) is a common liver condition caused by long-term alcohol consumption, and its specific molecular mechanism remains unclear. It may be influenced to some extent by ferroptosis and *Porphyromonas gingivalis* (*P.g*), which is an important pathogen of periodontitis.

**Materials and methods:**

C57BL/6 J mice and AML12 cells were selected as the study subjects. The periodontitis model was induced using *P.g*, and the alcoholic liver model was created. Pathological analysis was performed on the liver, intestine, and periodontal tissues. 16S rRNA sequencing was used to analyze changes in the intestinal flora and intestinal gap junction protein (zonula occludens-1 (ZO-1) and occludin) levels in each group. Ferroptosis indices were detected in the liver tissues and AML12 cells.

**Results:**

Oral exposure to *P.g* induced mice periodontitis and exacerbated alcohol-related liver injury. Both alcohol and *P.g* caused intestinal flora disturbance, damage to the intestinal epithelial barrier, increased permeability, and activation of mouse hepatocyte ferroptosis. Furthermore, *P.g* aggravated such alcohol-induced liver damage.

**Conclusion:**

Both alcohol and *P.g* can lead to intestinal flora disturbance, damage to the intestinal epithelial barrier, increased permeability, and the activation of mouse hepatocyte ferroptosis, and *P.g* can aggravate such alcohol-induced liver damage. Acyl-CoA synthetase long-chain family member 4 (ACSL4) and heme oxygenase-1 (HO-1) play important roles in the exacerbation of alcoholic liver injury by *P.g*.

## 1 Introduction

Alcoholic liver disease (ALD) may be influenced to some extent by periodontitis. Chronic periodontitis, a persistent inflammatory disease, is characterized by progressive alveolar bone loss, gingival recession, pocket formation in periodontitis, and, eventually, tooth mobility and loss. The progression of periodontitis is closely related to the subgingival microbial community and immune inflammation disorders (Bostanci and Belibasakis, 2012). Many studies have confirmed that periodontitis is closely associated with the progression of systemic diseases, including pancreatic cancer, colorectal cancer, atherosclerosis, and various liver diseases (Fan et al., 2018; Rubinstein et al., 2019; Kocgozlu et al., 2009; Nakahara et al., 2018; Chen et al., 2020). Lipopolysaccharides derived from oral bacteria may potentiate liver inflammation by binding to toll-like receptor (TLR)-4, triggering the upregulation of CD80 and CD86 co-stimulatory molecules, generating increased circulating matrix metalloproteinases, and activating T cells (Muñoz et al., 2014; Deleon-Pennell et al., 2013). Porphyromonas gingivalis (*P.g*), a member of the “red complex,” is an important pathogen of periodontitis. Some researchers have proposed and demonstrated that *P.g* is an important factor in the progression of non-alcoholic fatty liver fibrosis (Yao et al., 2023). In addition, patients with ALD often exhibited bacterial infections, and plasma levels of IgG, IgA, and IgM of *P.g* strains W83 and 33,277 were found to be higher in patients with acute alcoholic hepatitis (Zhou et al., 2019). ALD may be influenced by bacteria associated with periodontitis, as suggested by these findings.

Alcohol abuse is often associated with negative effects on periodontal health and the balance of oral flora. Alcohol consumption can disrupt the oral environment by altering pH levels and decreasing saliva secretion. Studies have shown that individuals with alcohol-dependent periodontitis experience a more severe form of periodontal disease and revealed that the oral microbiota of the Chinese drinking population and proposed that the *α* diversity and structure of the oral microbiota in drinkers were significantly altered. Moreover, the researchers found that oral health periodontal parameters were worse in both alcoholics with and without cirrhosis compared to non-alcoholics and healthy controls, and alcoholics had a lower total number of teeth (Novacek et al., 1995). Therefore, alcohol consumption is both a critical determinant in the development of ALD and a subject of interest for those studying periodontal health. There is a close relationship between ALD and periodontitis.

*P.g* may influence the progression of ALD through the oral–gut–liver axis and promote ferroptosis in liver cells. ALD is a common liver condition caused by chronic alcohol consumption. Recent research indicates that dysbacteriosis and the gut–liver axis contribute significantly to the development of ALD, in addition to the known metabolic role of ethanol and its derivatives (Dukić et al., 2023). Ethanol consumption can damage the intestinal defense barrier, trigger the release of endotoxin, and cause changes in intestinal flora composition and bile acid metabolism (Yan et al., 2023). Furthermore, as a conduit connecting the external environment to the gastrointestinal tract, oral microorganisms could potentially impact the gut microbiome. The overwhelming majority (over 50%) of microbial species regularly detected in healthy individuals’ mouths, such as *Streptococcus* and *Vibrio E. coli*, are also prevalent members of the gut microbiota, providing compelling evidence for the oral-to-gut translocation of these microorganisms (Yachida et al., 2019). Research has revealed the abnormal overgrowth of typical oral bacteria, such as *Streptococcus*, in the bowels of individuals diagnosed with irritable bowel syndrome (Yachida et al., 2019; Schenkein et al., 2000). Therefore, the oral–gut–liver axis may play a role in influencing the progression of ALD, potentially through *P.g*. It has been proposed that *P.g* aggravates ALD by exacerbating intestinal microbial metabolic disorders in alcohol-fed mice, which may depend on the activation of ferroptosis in hepatocytes (Yao et al., 2024). The specific mechanism through which *P.g* impacts the development of ALD remains somewhat of a mystery and requires further investigation.

In this study, we constructed *in vivo* and *in vitro* models of *P.g* stimulation and alcoholic liver disease and confirmed that *P.g* induced ferroptosis in hepatocytes via the oral–gut–liver axis and explored the mechanism. We assessed periodontal pathology, liver function, intestinal damage, intestinal microbiome changes, and ferroptosis in liver tissue.

## 2 Materials and methods

### 2.1 Culture and collection of *P.g*

*P.g* (ATCCBAA-308, BeNa Culture Collection) was activated on Columbia blood agar plates (BNCC352241, BeNa Culture Collection). The bacteria on the plate were cultured at 37°C under anaerobic conditions for 48-72 h. Then, the bacteria were collected with PBS to create a bacterial suspension and washed three times at 3000 rpm for 5 min. The amount of the bacteria culture was estimated by measuring absorption using a spectrophotometer at a wavelength of 600 nm (OD_600_). The bacterial suspension with an OD_600_ = 0.5 was used.

### 2.2 Animals model

**Figure 1:**
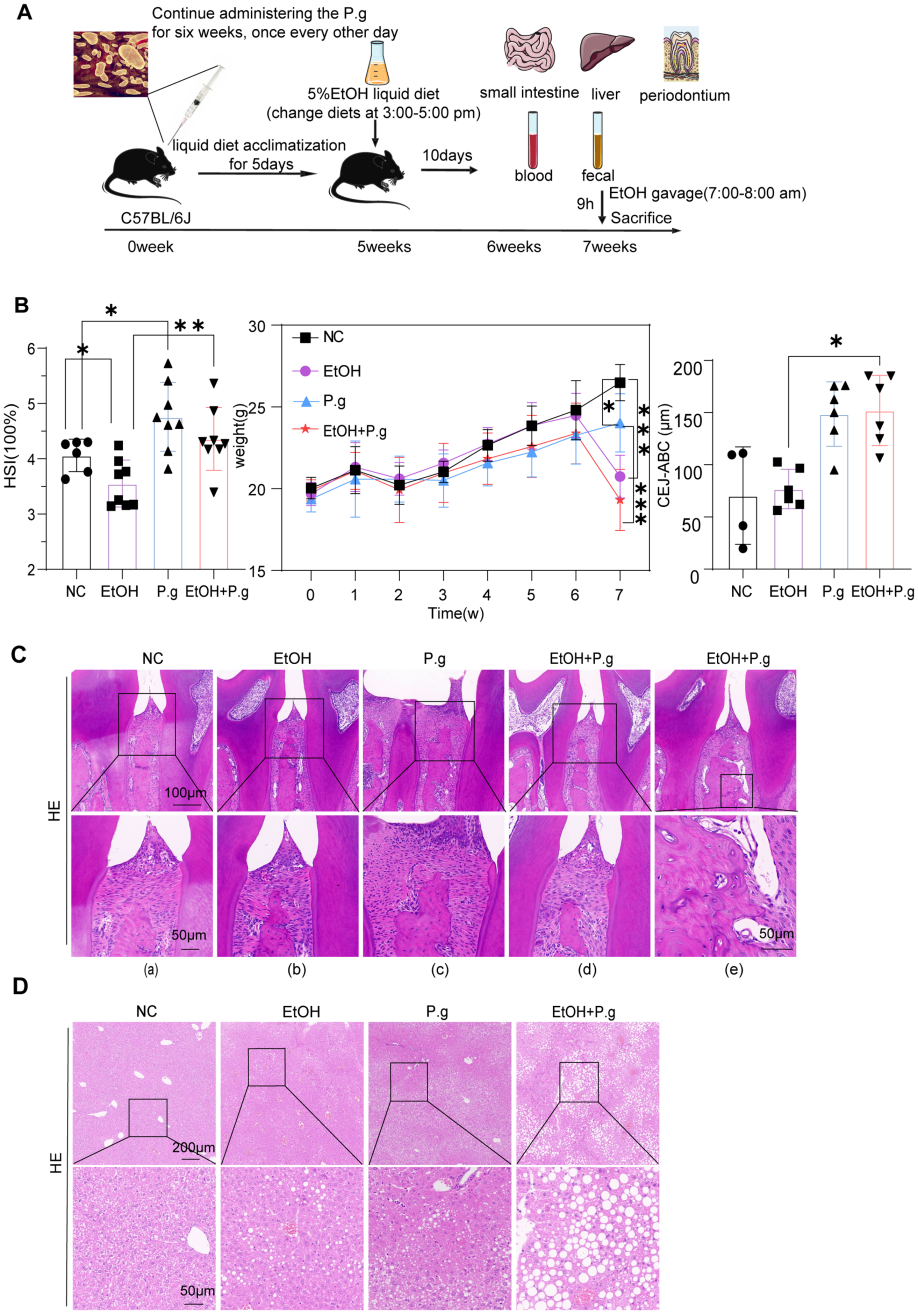
Oral exposure to *P.g* induced periodontitis in the mice and exacerbated alcohol-related liver injury. **(A)** Schema chart. **(B)** Hepatosomatic ratio, body weight, and cement-to-enamel junction–alveolar bone crest (CEJ–ABC) measurements in each group. **(C)** HE staining of the mandible section. Scale bars: 100 μm and 50 μm, *n*=6. **(D)** HE staining of the liver section. Scale bars: 200 μm and 50 μm, *n*=6. p^*^< 0.05, *p* ^**^< 0.01, and *p* ^***^< 0.001.

Five-week-old male C57BL/6 J mice (*n* = 30) were purchased from Changzhou Cavens Laboratory Animal Co., Ltd. Animal ethics approval was granted by the Clinical Medical Research Ethics Committee of the First Affiliated Hospital of Anhui Medical University. The study was approved by the research ethics committee at the institution where the research was conducted. The animal experiments were approved by the Animal Care and Use Committee of the First Affiliated Hospital of Anhui Medical University (No. 2022076). The mice were kept in adaptive feeding for 1 week in a barrier facility with strict control of an alternating 12-h light/dark cycle. All of the mice were randomly divided into four groups— a blank control group named NC (six mice), an ethanol group named EtOH (eight mice), a *P.g* group named P.g (eight mice), and an ethanol + *P.g* group named EtOH+P.g (eight mice). The classic NIAAA model was used to establish the alcoholic liver injury model in the mice. The mice were fed a liquid diet containing 5% alcohol (by volume) for 10 days, following 5 days of liquid diet adaptation. Fresh feed was changed daily between 3 and 5 pm. On the 11th day, a dose of 5 g/kg of high-concentration alcohol was administered by gavage. The experimental endpoint was reached 9 h after the gavage in the model group (Bertola et al., 2013). The mice in the P.g and EtOH+P.g groups were orally inoculated with 100 ul of suspended 2% carboxymethyl cellulose containing 1 × 10^9^ CFUs of *P.g*. After each inoculation, the mice were deprived of food and water for 2 h, once every other day, for 6 weeks. The mice were anesthetized with 100 mg/kg of 3% sodium pentobarbital. After confirming that the mice were completely unconscious, they were sacrificed by cervical dislocation. Blood, liver, stool, intestinal, and mandibular samples were then collected from the mice. As Figure 1A indicates, the animal model was designed using a construction diagram.

### 2.3 Fecal sample collection and analysis

Fecal samples from the mice were collected 2 days before the sacrifice, placed in an ice box, and promptly transferred and stored at −80°C in a freezer. Then, 16S rRNA sequencing was performed to analyze the intestinal microbiota. The MagPure Soil DNA LQ Kit (Magan) was used to extract genomic DNA from the samples. For bacterial diversity analysis, the V4-V5 variable region of the 16S rRNA gene was amplified using universal primers 515F (5’-GTGCCAGCMGCCGCGG-3′) and 907R (5′ -CCGTCAATTCMTTTRAGTTT-3′). The amplicon quality was visualized using agarose gel electrophoresis. The PCR products were purified using AMPure XP beads (Agencourt) and amplified for another round of PCR. After being purified with the AMPure XP beads again, the final amplicon was quantified using the Qubit dsDNA Assay Kit (Thermo Fisher Scientific, USA). The concentrations were then adjusted for sequencing. Sequencing was performed on an Illumina NovaSeq 6,000 with 250 bp paired-end reads. (Illumina Inc., San Diego, CA; OE Biotech Company; Shanghai, China). QIIME2 was used for alpha and beta diversity analysis.

### 2.4 Histology

The left lobe of the liver, small intestine, and mandible tissues from the mice were fixed with 4% paraformaldehyde, and histopathological changes in each group were observed using hematoxylin–eosin (HE) staining and Oil Red O staining. Paraffin embedded tissues (after mandibular decalcification) were fixed and sectioned (thickness 4 μm). Then, the sections were deparaffinized in xylene, rehydrated in gradient alcohol, and stained with hematoxylin and eosin (Wuhan Servicebio Technology CO., LTD). Moreover, the fixed frozen liver slices were rinsed with water and dried, placed in Oil Red O dye for 8-10 min, and then immersed in 60% isopropyl alcohol for differentiation. The slices were soaked in pure water for hematoxylin staining (Wuhan Servicebio Technology CO., LTD). Pathological diagnoses of the liver, intestine, and mandible were assessed by two specialist physicians and photographed under a microscope.

### 2.5 Immunohistochemistry (IHC)

The liver and intestinal tissues of the mice were treated with IHC. After the paraffin sections were dewaxed, antigen repair was carried out, followed by washing with PBS three times. Then, the slices were placed in a 3% H_2_O_2_ solution, incubated at room temperature for 20 min away from light, and washed with PBS three times. These sections were blocked with 3% rabbit serum or BSA at room temperature and incubated overnight at 4°C with the following primary antibodies: acyl-CoA synthetase long-chain family member 4 (ACSL4) antibody (1:200, 22,401-1-AP), heme oxygenase-1(HO-1) antibody (1:200, 10,701-1-AP), solute carrier family 7 member 11 (SLC7A11) antibody (1:200, PA1-16893), and 4-Hydroxynonenal (4-HNE) antibody (1:200, MA5-27570) for the liver tissue; occludin (1:400, GB111401, Servicebio) and zonula occludens-1 (ZO-1) (1:100, AF5145, Affinity) for the intestine tissue. After incubating with the secondary antibody at room temperature for 50 min, the color was developed using a DAB kit (DA1016, Beijing Solarbio Science &Technology Co., Ltd). The images were captured using the Panorama Tissue Quantitative Analysis System TissueFAXS Plus S and quantified using the ImageJ software.

### 2.6 Serum sample collection and analysis

Blood was collected from the mice into 1.5 mL EP tubes by eye transfusion and left at room temperature for 1 h. After blood clotting contractions, the samples were centrifuged at 3500–4000 rpm for 10 min. The supernatant was then collected and placed in clean centrifuge tube. Serum alanine aminotransferase (ALT), aspartate aminotransferase (AST), and serum iron (Fe) levels were determined using a fully automated biochemical analyzer (3,100, HITACHI), and the data were analyzed statistically.

### 2.7 Quantitative real-time PCR (qPCR)

AML12 cells, with a density of 1 × 10^6^ cells/well, were stimulated with anhydrous ethanol for 48 h and co-cultured with 26.7%-*P.g* supernatant for 24 h in 6-well plates. Total RNA was extracted from the liver and intestines of the mice models, as well as from the AML12 cells, using TRIzol®R reagent according to the manufacturer’s instructions (LIFE). The RNA samples were detected using the Nanodrop one (2505) system (Thermo Scientific, USA) with the A260/A280 absorbance ratio. We used the PrimeScript RT reagent Kit (TaKaRa, Dojindo, Kumamoto, Japan) to reverse transcribe the qualified RNA samples to obtain cDNA templates. The two-step method was performed under the following conditions: Stage I: 2 min at 42°C and infinite at 4°C; stage II: 10 min at 25°C; 30 min at 50°C; 5 min at 85°C; and infinite at 4°C. The qPCR reaction was amplified using TB Green® Premix Ex Taq™ II (Tli RNase H Plus) (TaKaRa, Dojindo, Kumamoto, Japan) in ABI QuantStudio6 Flex (Thermo Fisher Scientific, USA).

Amplification and detection were performed under the following optimal conditions: a hot start at 95°C for 1 min during the holding stage; the cycle stage consisted of 40 cycles at 95°C for 10s, 60°C for 30s, and 72°C for 1 min; and the melt curve stage involved 95°C for 15 s, 60°C for 1 min, and 60°C for 15 s. The comparative 2^−ΔΔCt method was used to calculate relative gene expression. The average Ct value of the target gene was normalized to the average Ct value of GAPDH to generate the ΔCt value, which was further normalized to the control sample to yield the ΔΔCt value. Each measurement was evaluated in triplicate. The gene expression rates from three independent experiments were expressed as mean ± SD. The primers used in this method are shown in Table 1.

**Table 1:** The primers of quantitative real-time PCR.

Name	Forward primer sequence (5′-3′)	Reverse primer sequence (5′-3′)	Species
ACSL4	TGGCTCATGTGCTGGAACTGAC	CAATCACCCTTGCTTCCCTTCTTG	Mouse
SLC7A11	TCATGTCCACAAGCACACTCCTC	AGAAGAGCATCACCATCGTCAGAG	Mouse
HO-1	AAGACCGCCTTCCTGCTCAAC	TCTGACGAAGTGACGCCATCTG	Mouse
Occludin	TGGCTATGGAGGCGGCTATGG	AAGGAAGCGATGAAGCAGAAGGC	Mouse
ZO-1	ACCCGAAACTGATGCTGTGGATAG	GCTGGCTGGCTGTACTGTGAG	Mouse
Claudin-1	GCTGGGTTTCATCCTGGCTTCTC	CCTGAGCGGTCACGATGTTGTC	Mouse
GAPDH	TCAAGAAGGTGGTGAAGCAG	AGGTGGAAGAATGGGAGTTG	Mouse

### 2.8 Cell culture

The AML12 cells (CatNo.GNM42) were provided by the Stem Cell Bank, the Chinese Academy of Sciences. The AML12 cells were cultured in DMEM (Thermo Scientific, USA) containing 10% fetal bovine serum (Gibco, USA), 100 U/mL penicillin, and 100 mg/L streptomycin at 37°C in a 5% CO_2_ humidification atmosphere. The AML12 cells, at a density of 1.2 × 10^5 cells per well, were placed in a 12-well plate. Experimental stimulation, including ferrostatin-1 (Fer-1, sigma, GER), ethanol, and *P.g* supernatant, was initiated when the degree of cell fusion reached 50–60%. The bacterial solution (OD_600_=0.5) was centrifuged in a high-speed (low-temperature) centrifuge at 4°C, 12000 rpm for 10 min, and the 26.7%-*P.g* supernatant was taken for use. The supernatant was then collected for use. For the EtOH and EtOH+P.g groups, the AML12 cells were stimulated with a complete culture medium containing 30ul of anhydrous ethanol for 48 h and then cultured with the 26.7%-*P.g* supernatant for 24 h in the P.g and EtOH+P.g groups. The total volume of the mixed culture components was 1500ul, consisting of 500ul of the *P.g* culture supernatant and 1000ul of the complete culture medium. The control group was cultured with the complete cell culture medium. Notably, the cells were treated with or without 2 μM Fer-1 for 48 h. The design of the cell experiment groups was similar to that of the animal groups.

For apoptosis detection, adherent cells were first collected using 0.25% trypsin without EDTA, then stained with an AnnexinV kit (BB-4101, Shanghai Beibo Biotechnology Co., Ltd) according to the manufacturer’s instructions. The apoptotic cells were detected using flow cytometry. The Mito-FerroGreen staining kit (Dojindo, Kumamoto, Japan) was used according to the manufacturer’s instructions. Images were captured using the CarlZeiss LSM880 fluorescence microscope.

### 2.9 Statistical analysis

IBM SPSS Statistics 26 was used for statistical analysis. Normal distribution tests were performed first when the data from each group were compared. The t-test was used for the data with a normal distribution, and the Mann–Whitney U test was used for the data with a non-normal distribution. The probability value, *p* <0.05, was considered statistically significant.

## 3 Results

### 3.1 Oral exposure to *P.g* can induce periodontitis in mice and exacerbate ALD

After the mice modeling process, all the mice across the four groups survived, but there were significant differences in weight and various other indices. The body weight of the mice in the EtOH and EtOH+P.g groups was significantly lower than that of the mice in the NC and P.g groups under the influence of alcohol, respectively (*p* < 0.001, Figure 1B). The effect of *P.g* on the body weight of the mice could not be ignored. The body weight of the mice in the P.g group was significantly lower than that of the mice in the NC group (*p* < 0.05, Figure 1B), and the body weight of the mice in the EtOH+P.g group was also lower than that of the mice in the EtOH group. The hepatosomatic ratio was lower in the P.g (*p* < 0.05, Figure 1B) and EtOH+P.g (*p* < 0.001, Figure 1B) groups than in the NC and EtOH groups, respectively.

To show the effect of *P.g* on the periodontal tissue in the mice, the cement-to-enamel junction–alveolar bone crest (CEJ–ABC) was used to evaluate alveolar bone absorption. The EtOH+P.g group showed more obvious absorption (*p* < 0.05, Figure 1B). The HE stained sections of the mandible showed the following pathological changes in the EtOH+P.g and P.g groups: gingival recession, elongation of the epithelial pegs [Figure 1C(c)], presence of bone resorption lacunae in the alveolar bone [Figure 1C(e)], disappearance of basophilic edges at the crest of the alveolar bone ridge [Figure 1C(c–e)], visibility of large fiber bundles above it, an obvious necrotic area in the gingival col. [Figure 1C(d,e)], and disorganized fiber arrangement within the periodontal membrane in the P.g and EtOH+P.g groups [Figure 1C(c–e)]. Furthermore, the HE staining showed that the EtOH+P.g group had more severe inflammatory infiltration and hepatic steatosis after the modeling procedures compared to the EtOH and P.g groups (Figure 1D).

These results demonstrated that the animal model of alcoholic liver injury with periodontitis was successfully constructed and that *P.g* caused periodontal tissue inflammation in the mice and aggravated alcohol-induced liver injury.

### 3.2 *P.g* and alcohol consumption can result in significant alterations in the gut microbiota

**Figure 2:**
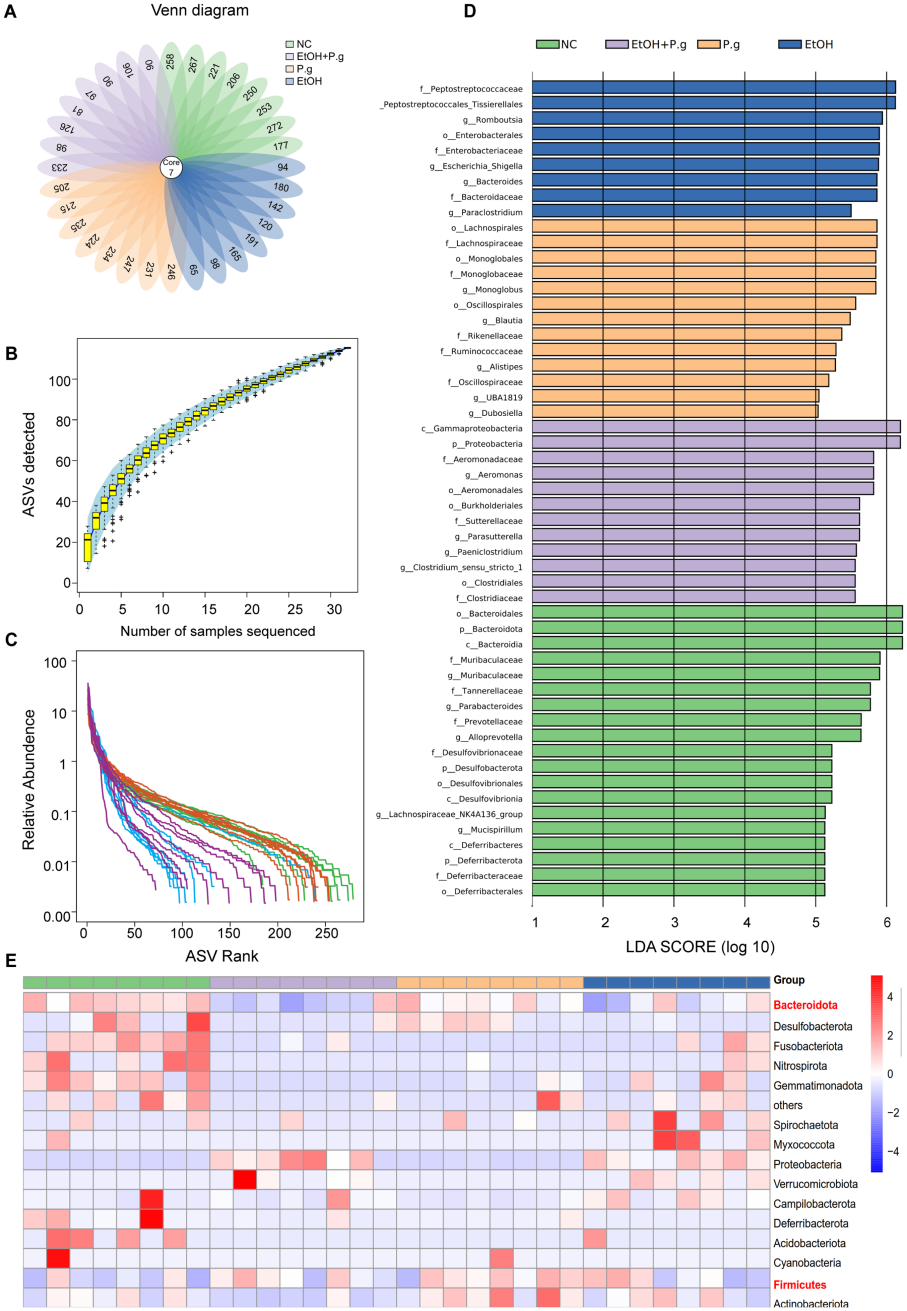
Differential analysis of the intestinal microbiome in the mice. **(A)** Venn diagram. **(B)** Sparse curve showing reasonable sequencing depth. **(C)** Rank abundance curve. **(D)** LefSe analysis (LDA=10). **(E)** A heatmap was used to show the top 15 distinct species at the phylum level. p^*^< 0.05, *p* ^**^< 0.01, and *p* ^***^< 0.001.

**Figure 3:**
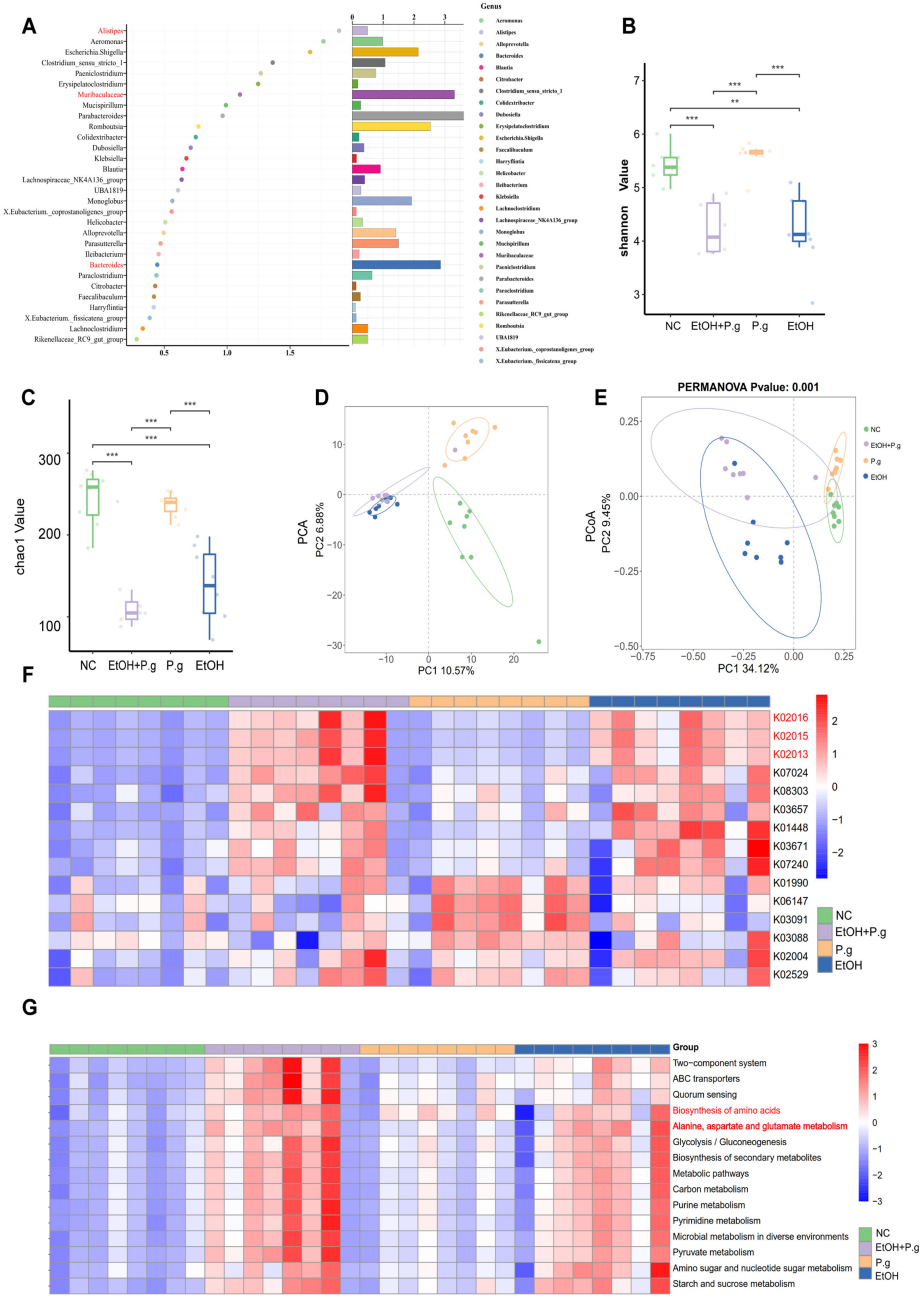
16S rRNA analysis of the intestinal microbiome in the mice. **(A)** The random forest model identified ASVs, *n*=8. **(B,C)** Alpha diversity analysis of the species, *n*=8. **(D)** Principal component analysis (PCA) of the gut microbiota from the four groups, *n*=8. **(E)** Principal Coordinates Analysis (PCoA) of the gut microbiota from the four groups using the unweighted and weighted UniFrac distances, *n*=8. **(F)** PICRUSt2 analysis to predict functional abundance, *n*=8. **(G)** KEGG function prediction, *n*=8. p^*^< 0.05, *p* ^**^< 0.01, and *p* ^***^< 0.001.

After analyzing 32 fecal samples from the mice in the four groups, the sequencing results showed that the number of differential ASVs was 287 (Figure 2A). The sparse curve showed reasonable sequencing depth (Figure 2B). The rank abundance curve showed that the curves of the EtOH and EtOH+P.g groups decreased rapidly, indicating that dominant strains appeared in its samples and inhibited the growth of other microorganisms (Figure 2C). Compared to other groups, Bacteroidetes and Firmicutes were predominant in the NC group. However, the abundance of Bacteroidetes in the feces of the mice treated with ethanol and *P.g* decreased significantly, while the level of Proteobacteria increased. The heatmap illustrated the top 15 distinct species at the phylum level and showed significant differences among the groups (Figure 2E). The LefSe analysis (LDA=10) showed that the species differences among the groups were mainly *Peptostreptococcus* in the EtOH group, *Bacteroides* in the NC group, and *Gammaproteobacteria* and *Prevotella* in the EtOH+P.g group (Figure 2D). It is worth noting that *Prevotella* is related to intestinal inflammation. The *α* diversity analysis showed that under the effect of ethanol and *P.g*, the community richness and diversity of the intestinal flora were significantly reduced. The Shannon and Chao1 indices in the NC and P.g groups were significantly higher than those in the alcohol groups (*p* < 0.001, Figures 3B, 3C). In addition, the Chao1 index in the P.g group treated with bacteria was also lower than that in the NC group, although this was not statistically significant (Figure 3C). Principal coordinate analysis (PCoA) was used to detect the fecal microbiota of the four groups based on the Bray–Curtis distance. Unweighted and weighted UniFrac distance PCoA of the samples with significant differences after the alcohol treatment showed that alcohol consumption had significant effects on the intestinal microbiota composition in the mice (Figure 3E). The principal component analysis (PCA) showed that both oral *P.g* and ethanol had a significant effect on the microbiota composition in the mice (Figure 3D). The consumption of ethanol and *P.g* led to significant changes in the gut microbiome of the mice, which is in line with previous studies (Kong et al., 2021).

### 3.3 The effects of *P.g* and alcohol intake on the gut microbiome of the mice may be related to ferroptosis

ASVs were identified using the random forest model, and we noticed the role of Alistipes, Muribaculaceae, and Bacteroides in the intestinal microbiota (Figure 3A). The PICRUSt2 functional prediction based on 16S rRNA sequencing revealed significant alterations in the iron transport systems (*K02016*, *K02013*, and *K02015*), particularly in the ethanol-exposed groups (Figure 3F). In addition, we observed that KEGG level 2 pathways, such as amino acid metabolism, lipid metabolism, and carbohydrate synthesis, were enriched in the EtOH and EtOH+P.g groups but reduced in the P.g group. KEGG level 3 pathways, such as the biosynthesis of amino acids and the metabolism of alanine, aspartic acid, and glutamate, were reduced in the P.g group but enriched in the EtOH and EtOH+P.g groups (Figure 3G). It is worth noting that differences in ferric transporters, disruption of amino acid and lipid synthesis, and glutamate and glutamine biosynthesis are strongly linked to ferroptosis (Yao et al., 2024).

Therefore, these results suggested that the intake of ethanol and *P.g* not only led to significant changes in the gut microbiome but was also associated with ferroptosis in the tissue cells.

### 3.4 *P.g* and alcohol can impair the function of the intestinal barrier

**Figure 4:**
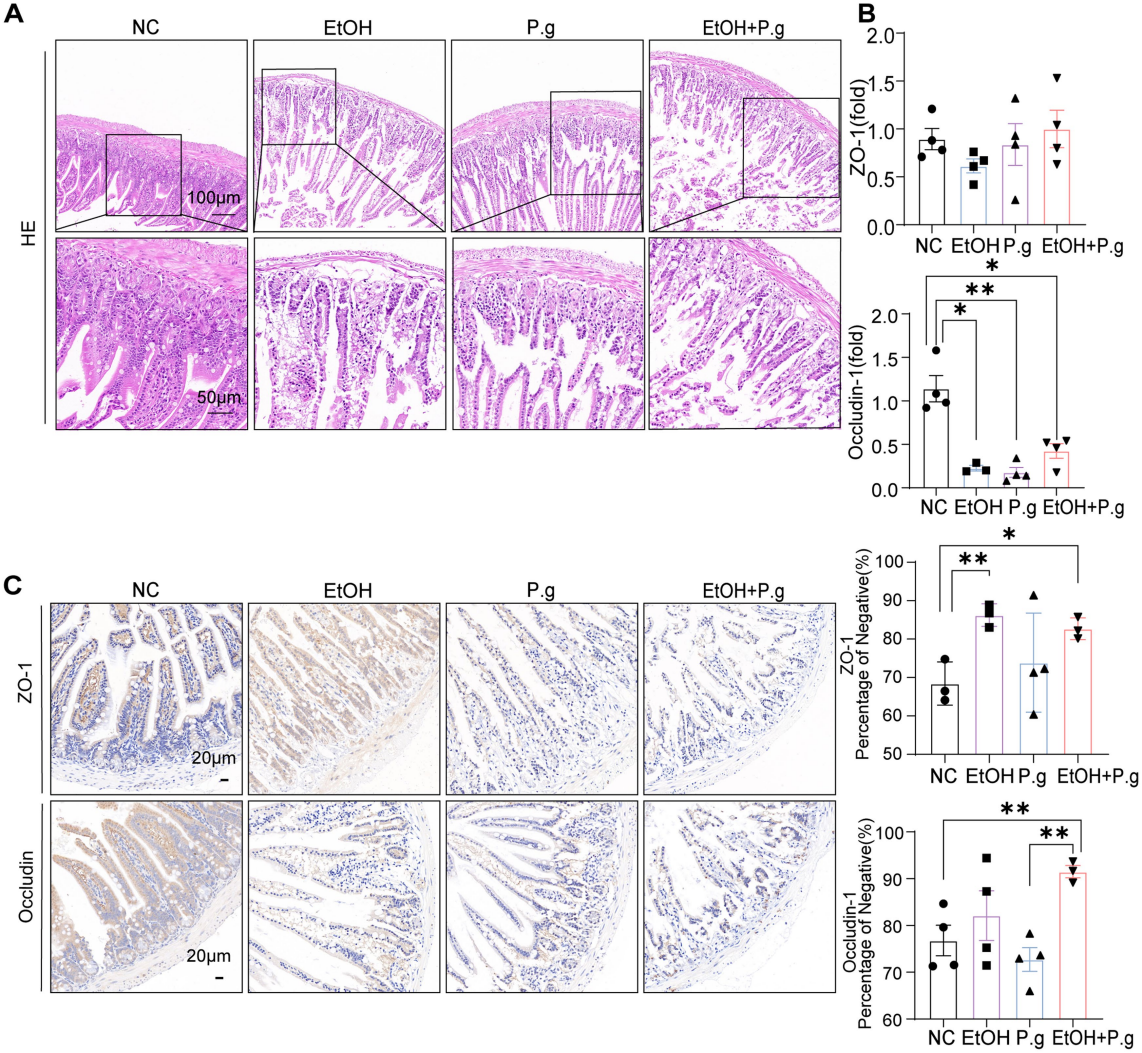
Damage to the intestinal tissue caused by *P.g* and alcohol in the mice. **(A)** HE staining of the small intestine section. Scale bars: 100 μm and 50 μm, *n*=6. **(B)** Histogram of the mRNA qPCR expression of ZO-1 and occludin in the small intestine, *n*=3. **(C)** Immunohistochemistry of ZO-1 and occludin in the small intestine, quantified using densitometry. Scale bar: 20 μm, *n*=3. p^*^< 0.05, *p* ^**^< 0.01, and *p* ^***^< 0.001.

The HE sections of the small intestine in the mice showed that the intestinal mucosal structure of the mice in the EtOH, P.g, and EtOH+P.g groups was damaged, the small intestinal villi were destroyed, and extensive tissue damage penetrated the intestinal wall. The wall of the small intestine became thinner under the effects of ethanol and *P.g* (Figure 4A). In addition, ethanol and *P.g* resulted in a marked reduction in the expression of genes involved in the intestinal epithelial tight junction barrier, including occludin (*p* < 0.05, Figure 4B) and ZO-1, as shown by qPCR analysis. Meanwhile, this situation was verified using immunohistochemistry, and the expression of occludin and ZO-1 in the EtOH, P.g, and EtOH+P.g groups was significantly lower than that in the NC group (Figure 4C). It can be seen that *P.g* and alcohol can impair intestinal barrier function.

### 3.5 The oral administration of *P.g* has the potential to aggravate alcoholic liver injury *in vivo* through the process of ferroptosis

**Figure 5:**
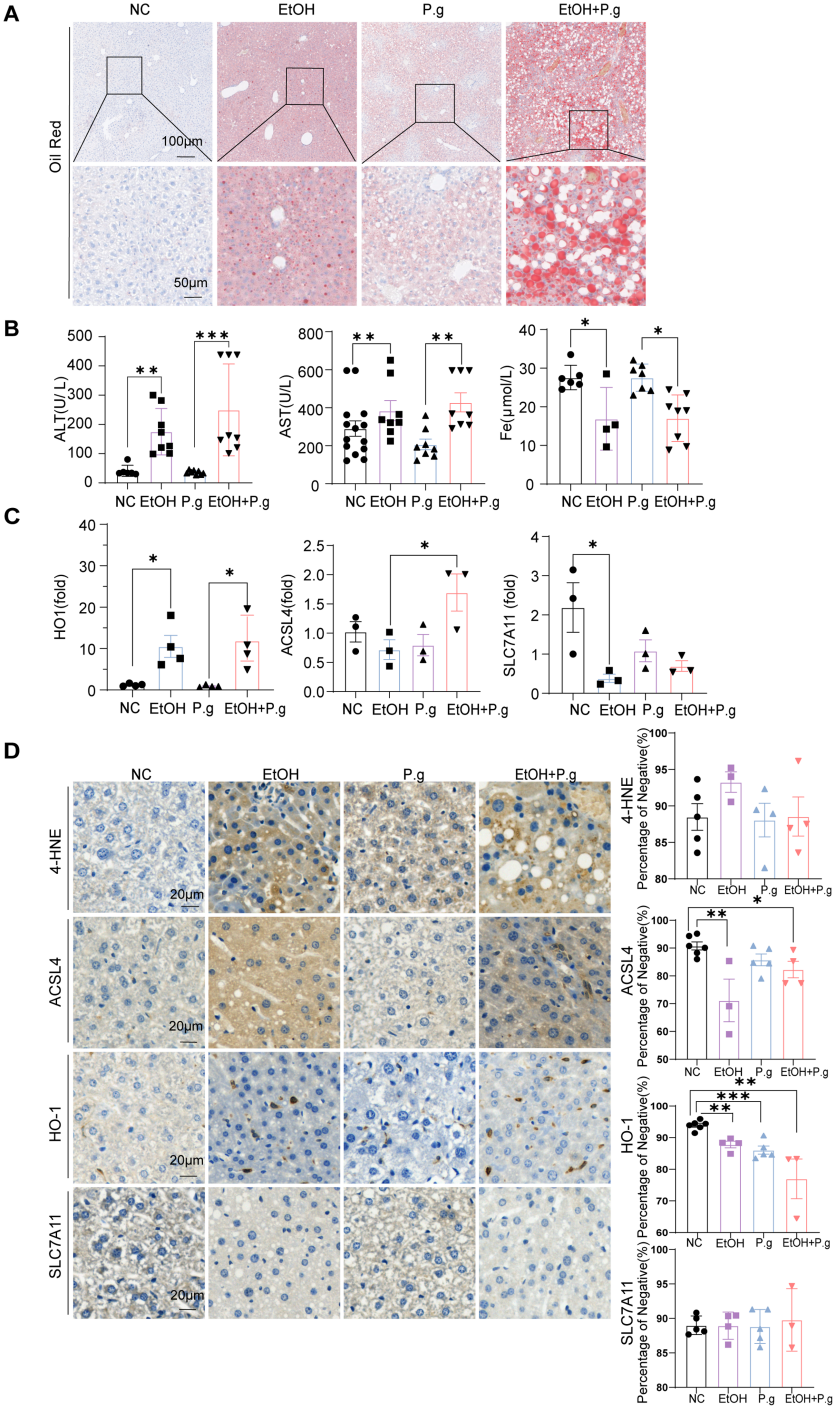
*P.g* aggravated alcoholic liver injury *in vivo* via ferroptosis. **(A)** Oil Red O staining of the liver section. Scale bars: 100 μm and 50 μm, *n*=6. **(B)** Serum biochemical indices of the mice, *n*=6. **(C)** Histogram of the mRNA qPCR expression of HO-1, ACSL4, and SLC7A11 in the liver of the mice, *n*=3. **(D)** Immunohistochemistry of 4-HNE, ACSL4, HO-1, and SLC7A11 in the liver of the mice, quantified using densitometry. Scale bar: 20 μm, *n*=6. p^*^< 0.05, *p* ^**^< 0.01, and *p* ^***^< 0.001.

The results of the liver Oil Red O staining confirmed that both *P.g* and alcohol intake could cause hepatic fatty degeneration, while the liver tissues from the mice in the EtOH+P.g group showed more serious changes, suggesting that *P.g* may apodictically aggravate alcoholic liver injury (Figure 5A). The extent of serum hepatocyte damage in the EtOH+P.g group, as indicated by elevated levels of ALT and AST, was greater than that observed in the mice exposed to either factor alone (*p* < 0.01, Figure 5B). This finding is consistent with the pathological pattern of liver tissue damage observed. According to the KEGG prediction results from the mice’s intestinal fecal sequencing, we also measured the relevant indices of iron metabolism in the mice serum. It was clear that alcohol consumption significantly reduced the serum iron levels in the EtOH and EtOH+P.g groups and that the disruption of iron metabolism was closely related to ferroptosis (*p* < 0.05, Figure 5B). Therefore, the mRNA expression levels of the ferroptosis-related genes ACSL4, HO-1, and SLC7A11 were gauged using qPCR. The results showed that the expression levels of ACSL4 and HO-1 were significantly increased in the EtOH, P.g, and EtOH+P.g groups, especially in the EtOH+P.g group, while the expression level of SLC7A11 was decreased in the EtOH and EtOH+P.g groups (*p* < 0.05, Figure 5C). The expression levels of ACSL4, HO-1, and SLC7A11 were evaluated using IHC, which confirmed the same expression levels observed by the qPCR (*p* < 0.05, Figure 5D). These findings indicated that ferroptosis activation occurred in the liver of the mice in both the EtOH and EtOH+P.g groups and that the changes were more obvious in the EtOH+P.g group, suggesting that oral *P.g* could exacerbate ferroptosis and pathological alteration in ALD.

### 3.6 Ferrostatin-1 (Fer-1) can relieve the ferroptosis caused by *P.g* aggravating alcohol-induced hepatocyte injury

**Figure 6:**
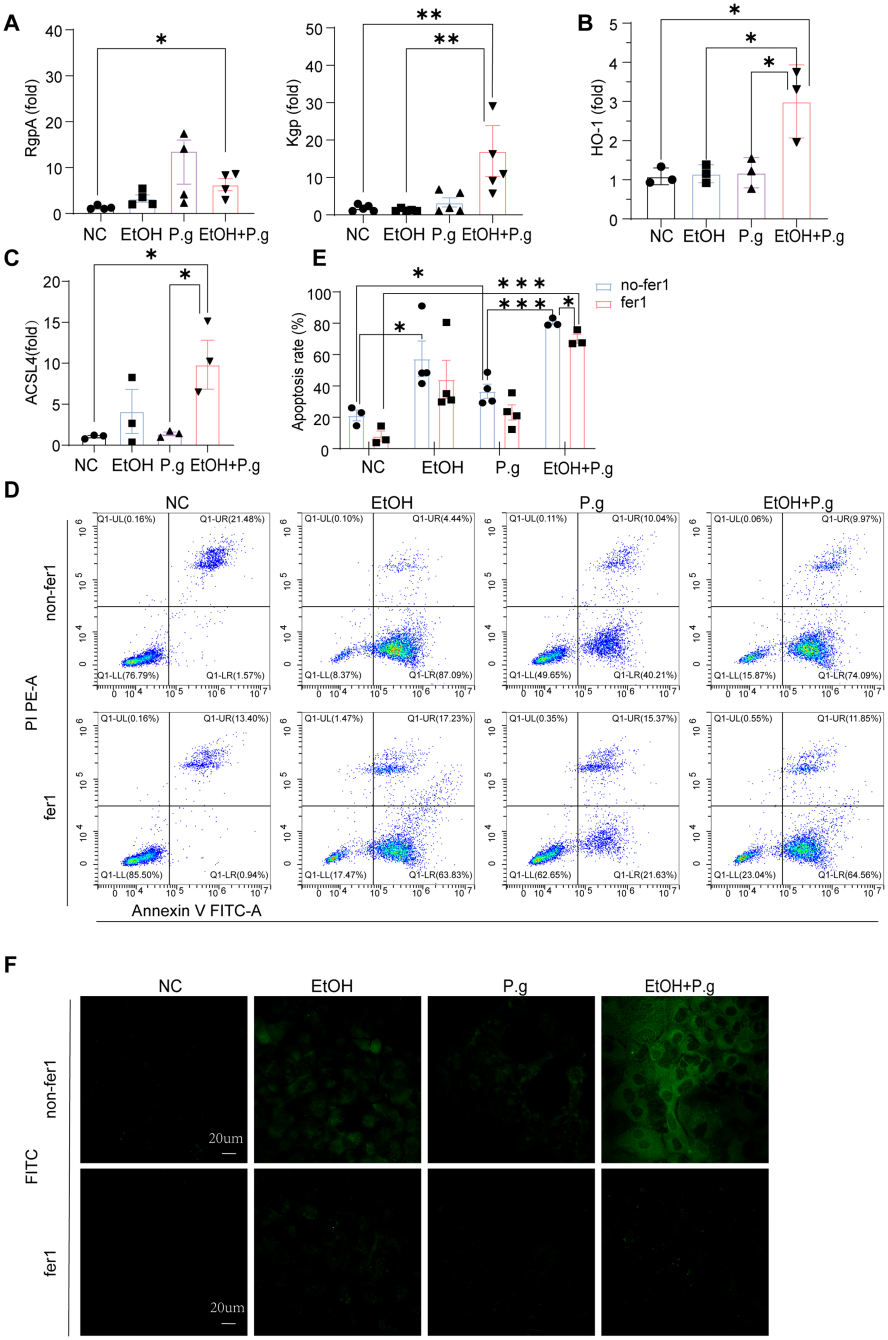
Fer-1 relieved the ferroptosis caused by *P.g* aggravating alcohol-induced hepatocyte injury *in vitro*. **(A–C)** Histogram of the mRNA qPCR expression of RgpA, Kgp, HO-1, and ACSL4 in the AML12 cells, *n*=3. **(D,E)** Flow cytometry exhibited apoptosis in the four groups, *n*=3. **(F)** Mito-FerroGreen staining of the ALM12 cells in each group, *n*=3. p^*^< 0.05, *p* ^**^< 0.01, and *p* ^***^< 0.001.

To further validate the occurrence of ferroptosis with alcohol and *P.g* exposure in liver cells, *in vitro* AML12 cell experiments were conducted. The qPCR results showed that the levels of gingipain K (Kgp) (*p* < 0.01, Figure 6A) and Arg gingipains (RgpA) (*p* < 0.05, Figure 6A) were significantly increased in the P.g and EtOH+P.g groups, indicating the effective impact of the bacteria on the cells. Moreover, the mRNA expression levels of ACSL4 and HO-1 were gauged using qPCR. A significant upregulation of ACSL4 and HO-1 expression was observed in the EtOH, P.g, and EtOH+P.g groups, with the EtOH+P.g group demonstrating the most dramatic change (*p* < 0.05, Figures 6B,C).

Subsequently, we alleviated this cellular damage with the ferroptosis inhibitor Fer-1. Flow cytometry demonstrated that all of the EtOH, P.g, and EtOH+P.g groups exhibited obvious apoptosis, which was, however, successfully mitigated by the ferroptosis inhibitor Fer-1 (2 μM) (*p* < 0.05, Figures 6D,E). In contrast, apoptosis was more obvious in the EtOH+P.g group (*p* < 0.05, Figure 6E). The Mito-FerroGreen staining showed that the fluorescence intensity of the P.g, EtOH, and EtOH+P.g groups was enhanced and that the fluorescence intensity of the EtOH+P.g group was more obvious. The ferroptosis inhibitor Fer-1 could alleviate this tendency (Figure 6F). It indicated that both *P.g* and alcohol can cause ferroptosis in hepatocytes and that *P.g* worsened alcohol-induced ferroptosis in hepatocytes *in vitro* experiments.

These results reached the same conclusion as the *in vivo* experiments, showing that *P.g* can aggravate ferroptosis caused by alcoholic hepatocyte injury *in vitro*.

## 4 Discussion

This study investigated the effect of *P.g*, a key pathogen of periodontitis, on ALD. Our findings propose that the oral colonization of *P.g* significantly promotes the progression of ALD. This effect is likely mediated by changes in the intestinal microbiota, subsequent metabolic disturbance, and ferroptosis.

Studies have shown that periodontitis is closely related to gut microbes (Cani, 2018). Oral and intestinal microbes may interact through the blood or digestive system, despite the presence of their own colonized flora (Schenkein et al., 2000). It has been demonstrated that *P.g* exacerbates ALD by altering the composition of the intestinal flora and the host immune response in mice (Gao et al., 2023). This phenomenon was also confirmed in our study, which showed that periodontitis occurred in the mice after oral exposure to *P.g* (Figure 1C). In addition, oral *P.g* aggravated alcoholic liver injury and increased fat formation in the mice with alcoholic liver injury (Figures 1C, 5A). After the oral administration of *P.g*, the liver cell function indicators ALT and AST were further upregulated (Figure 5B).

Recently, oxidative stress and alterations in the composition of the gut microbiome have been reported to contribute to the progression and severity of ALD (Szabo, 2015). In our study, intestinal microbiota disturbances also occurred in the mice. Exposure to ethanol can lead to harmful changes in the gut barrier, including endotoxin leakage from the gut microbiota. As shown above in our results, the consumption of *P.g* and alcohol can result in significant alterations in the gut microbiota. Consistent with previous studies (Do et al., 2022), our findings highlight that *P.g* and alcohol consumption causes alterations in gut microbiome composition, specifically by influencing the abundance of *Alistipes*, *Bacteroides*, and *Muribaculum* (Figure 5B). The change in the abundance of *Alistipes* and *Bacteroides* in the gut has been linked to elevated blood ALT levels, potentially contributing to the development of ALD (Li et al., 2021; Llopis et al., 2016). *Bacteroides* are bacteria that are well known for their ability to produce endotoxins and can significantly increase intestinal permeability (Xie et al., 2016). Colonization by intestinal *Prevotella* leads to changes in the metabolism of the microbiome, reducing the production of IL-18, which increases gut inflammation and may be relevant to liver disease (Cani, 2018). In addition, alcohol and *P.g* significantly reduced the levels of intestinal ZO-1 and occludin, molecules that play important roles in protecting intestinal permeability (Figures 3B,C). Therefore, these findings suggest that these bacterial changes lead to the breakdown of intestinal barrier function by increasing the levels of toxic substances in the gut and may worsen liver damage. Notably, previous studies have shown that *P.g* can aggravate colon inflammation through gut microbes (Gao et al., 2023). The damaging effects of alcohol and bacteria on the intestinal tract are not limited to the small intestine but may extend throughout the entire tract. Although *P.g* can be detected in intestinal tissue, inflammation in the colon is not directly triggered by *P.g* and can be related to the gut microbiota (Szabo, 2015). This means that the aggravation of alcoholic liver damage by *P.g* may also occur indirectly through changes in gut microbes and their metabolites, which need further research.

ALD is closely associated with both intestinal flora and ferroptosis. Recent studies have revealed that ferroptosis is a crucial mediator of inflammation triggered by microbial infection, and the underlying mechanism is due to competition for iron between the microorganisms and host cells (Tang et al., 2018). In our study, we showed that the serum iron in the mice was significantly reduced in the EtOH and EtOH+P.g groups (*p* < 0.05, Figure 5B). In addition, we observed that the iron complex transport proteins (K02016, K02013, and K02015) were significantly different (Figure 3F) across the four groups, as shown by the PICRUSt2 analysis. These results suggest that alcohol and *P.g* may disrupt the process of iron metabolism and utilization in mice through alterations in gut microbes and their metabolites, which are closely related to mitochondrial function (Chen et al., 2021). In fact, the liver is prone to oxidative damage, and excessive iron accumulation is one of the main characteristics of most liver diseases (Wang et al., 2017). In addition, our study showed that the expression levels of ACSL4 and HO-1 were increased in the EtOH, P.g, and EtOH+P.g groups, with a significant increase in the EtOH+P.g group (*p* < 0.05, Figures 5C,D). This indicated that ferroptosis activation occurred in the liver of the mice in both the EtOH and EtOH+P.g groups and that the changes were more obvious in the EtOH+P.g group, suggesting that oral *P.g* can exacerbate ferroptosis and pathological alterations in ALD. Researchers have also found that large amounts of ROS accumulation and lipid peroxidation caused by alcohol could be improved by using a ferroptosis inhibitor in both cell culture and a mouse model of ALD. Dimethyl fumarate inhibits ferroptosis by activating the nuclear factor erythroid 2-related factor 2 (Nrf2) pathway and has a protective effect on ethanol-induced oxidative damage (Guillot and Tacke, 2019). However, a deficiency of intestinal SIRT1 can normalize alcohol-induced iron imbalances and limit the occurrence of ferroptosis, enabling improvements in alcohol-induced liver damage (Zhou et al., 2020). Therefore, we suggest that *P.g* exacerbates ALD through intestinal microbiota-HO-1-ACSL4-dependent ferroptosis.

**Figure 7:**
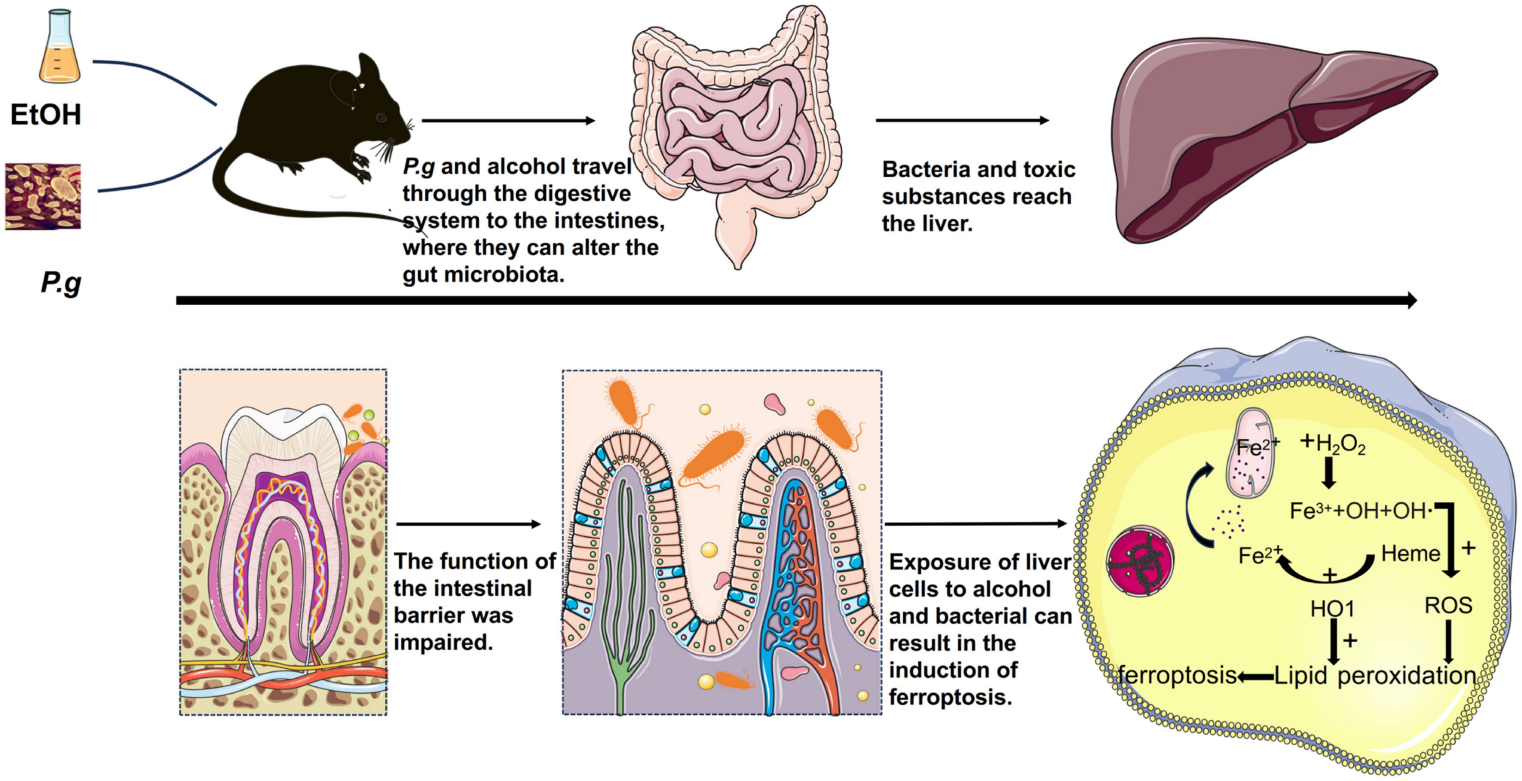
*P.g* aggravated alcohol-related liver injury via gut microbiome-HO-1-ACSL4-dependent ferroptosis. *P.g* and alcohol consumption resulted in considerable damage to the intestinal epithelial barrier in the mice. *P.g* may lead to the disturbance of iron metabolism and utilization in hepatocytes through the intestinal microbiota, resulting in excessive accumulation of Fe^2+^ in mitochondria and aggravating alcohol-related liver injury through ferroptosis. HO-1 and ACSL4 are involved in this process.

Next, we performed *in vitro* cell experiments with the ferroptosis inhibitor Fer-1 in each group for further research. The results of the flow cytometry confirmed that all experimental groups under the influence of alcohol and *P.g* exhibited significant apoptosis of the AML12 cells, especially the EtOH+P.g group. However, Fer-1 exhibited a partial reversal of this trend *in vitro* (Figures 6D,E), suggesting that *P.g* aggravated alcohol-related liver injury and induced ferroptosis. As we discussed earlier, the changes in iron metabolism in the mice may be related to mitochondrial function, and there were significant changes in the glutamate metabolic pathways in the experimental groups (Figures 3F,G). It has long been established that glutamate is necessary for cysteine deprivation-induced ferroptosis and that one of the main functions of glutamine breakdown is to promote the tricarboxylic acid cycle in mitochondria (Gao et al., 2019). Therefore, to detect the changes in iron ions within the mitochondria in each group, we conducted Mito-FerroGreen staining, which showed that the fluorescence intensity of the EtOH+P.g group was more obviously enhanced than that of the single-factor stimulation group. However, Fer-1 was able to alleviate this tendency (Figure 6F). Extracellular iron ions are absorbed by cells and can be introduced into mitochondria via SLC25A37 and SLC25A28. Fe^2+^ in mitochondria can be used to synthesize heme and Fe-S clusters or stored in mitochondrial ferritin. Conversely, excess mitochondrial Fe^2+^ mediates ROS production or leads to abnormal enzyme activity, and impaired mitochondrial iron metabolism leads to ferroptosis (Chen et al., 2021). Interestingly, it has been shown that heme can directly induce ferroptosis in primary neurons or human monocytes, and this process can be further regulated by cytoplasmic or mitochondrial HO-1 (Alim et al., 2019). It has been indicated that elevating HO-1 expression may lead to the release of iron ions during heme degradation, thereby augmenting hepatocyte ferroptosis (Liao et al., 2021). HO-1 is vital for cell physiology and provides antioxidant protection by breaking down heme into Fe^2+^, carbon monoxide, and biliverdin (Zhang et al., 2021). However, excessive heme breakdown can lead to Fe^2+^ overload, exacerbating oxidative stress and potentially causing cell damage, especially in the liver. Our study confirmed a significant accumulation of Fe^2+^ in the cell mitochondria through fluorescence detection (Figure 6F). Therefore, understanding the balanced expression of HO-1 may be important for maintaining normal physiological activities of cells. In addition, some studies have confirmed that a chain reaction of lipid peroxidation occurs during the peroxidation of polyunsaturated fatty acids, which produces a large number of ROS and destroys membrane phospholipids, thus promoting ferroptosis. In this process, ACSL4 preferentially recognizes the unsaturated fatty acid AA as a substrate and subsequently enhances cell sensitivity to ferroptosis by regulating the corresponding lipids (Wang et al., 2022). Our results showed a significant upregulation of ACSL4 and HO-1 expression in the EtOH, P.g, and EtOH+P.g groups, with the EtOH+P.g group demonstrating the most dramatic change. This further supports the conclusion that the effects may be caused by the HO-1 and ACSL4 pathways (Figures 6B,C).Therefore, we propose that *P.g* may lead to the disturbance of iron metabolism and utilization in hepatocytes through the intestinal microbiota, resulting in excessive accumulation of Fe^2+^ in mitochondria and aggravating ALD via ferroptosis. HO-1 and ACSL4 are involved in this process (Figure 7).

Our study has some limitations. Although we did not explicitly assess necrosis- or apoptosis-specific markers, the clear and significant protective effects of the selective ferroptosis inhibitor Fer-1 strongly suggest that ferroptosis is the primary mechanism of hepatocyte injury in our model. In addition, in the case of liver injury, apoptosis and necrosis can coexist or be secondary to ferroptosis. Due to the complexity of the intestinal flora and the possible interactions among various strains, the changes cannot be quantified with current research methods. Moreover, our study was conducted primarily in mice and hepatocytes, which may limit its immediate translational relevance to human patients. However, we believe that the mechanisms identified in our investigation, particularly the role of ferroptosis in the progression of ALD exacerbated by *P.g*, are highly relevant to human health. Currently, there are no effective therapeutic targets to halt the progression of ALD. This study investigated how *P.g* contributes to ALD progression, highlighting the involvement of HO-1 and ACSL4 in this process. These findings may provide therapeutic strategies for improving the treatment of ALD.

## 5 Conclusion

Both alcohol and *P.g* can cause disturbances in the intestinal flora, damage to the intestinal epithelial barrier, increased permeability, and activation of hepatocyte ferroptosis in mice. *P.g* can aggravate alcohol-induced liver damage. ACSL4 and HO-1 play important roles in the aggravation of alcoholic liver injury caused by *P.g*.

## Data Availability

Raw data are available on reasonable request. The datasets generated during the current study are uploaded in the NCBI (Accession number: PRJNA1193892).
